# Ranking hospitals on avoidable death rates derived from retrospective case record review: methodological observations and limitations

**DOI:** 10.1136/bmjqs-2015-004366

**Published:** 2015-07-03

**Authors:** Gary Abel, Georgios Lyratzopoulos

**Affiliations:** 1 Cambridge Centre for Health Services Research, Primary Care Unit, University of Cambridge, Cambridge, UK; 2 Department of Epidemiology & Public Health, University College London, London, UK

**Keywords:** Statistics, Health policy, Medical error, measurement/epidemiology, Performance measures

Reducing the number of avoidable deaths in hospital is the focus of many quality improvement initiatives worldwide.^1^ Comparing indicators of avoidable mortality between different hospitals could help to target improvement efforts, but optimally defining and measuring hospital deaths that could be deemed preventable remains a challenge.^2^ Unlike performance comparisons based on hospital standardised mortality ratio (HSMR), a new policy initiative announced by the UK Government will rank hospitals for avoidable mortality based on case reviews of 2000 deaths in English hospitals each year. Although this initiative aims to overcome limitations of current policies, two statistical properties of the proposed approach mean that it is unsuitable for classifying hospital performance.

The first issue relates to the ability to identify whether any one death really was avoidable on a case-by-case basis. It would appear^3^ that the planned process is based on work by Hogan *et al*^4^ using retrospective case record review (RCRR). In line with previous studies using RCRR, these investigators asked experienced clinicians to rate whether a death was preventable on a 6-point Likert scale.^4^ ^5^ Their study recognised that the use of a semicontinuous scale better reflects ‘the probabilistic nature of reviewers’ decision making more closely than requiring a simple “yes” or “no” response’.^4^ ^5^ However, in operationally defining an avoidable death, the probabilistic component of the instrument is lost because a fixed cut-off is used such that deaths where it is judged that there is more than a 50% chance that the death was preventable are classified as avoidable, and those below 50% are not. (It should be noted that the somewhat arbitrary choice of a 50% cut-off value is not the real issue here, but rather the dichotomisation itself is. However, hereafter, we assume a 50% cut-off value is used as proposed).^4^

By dichotomising cases into being avoidable or not, the information about the distribution is lost. One might naively argue that the probabilities above 50% will average out with those below 50% to give the right answer. In fact, this is only true when the mean chance of a death being avoided (where the chance is greater than zero) is 50%. This is a strong assumption that will nearly always be untrue. To illustrate this further, we can consider two scenarios. First, a scenario where there were 100 deaths, each with a 60% chance of preventability, implying that 60 deaths would have been avoided if there were no problems in care (assuming independence between cases); and another scenario where there were 100 deaths, each with a 20% chance of preventability, implying that 20 deaths would have been avoided. By only focusing on deaths where the judged preventability is greater than 50% (ie, the proposed operational definition of an avoidable death), we would have estimated 100 ‘avoidable deaths’ in the first scenario and zero ‘avoidable deaths’ in the latter—both conclusions being evidently untrue. These errors arise as a result of ignoring that there will be a range of risks that deaths are preventable. In reality, the distribution of risk that deaths are preventable is likely to be highly skewed, with many deaths where there is a small chance that it was preventable and a few deaths where the chance that the death was preventable is large. By ignoring this continuum of risk, only those deaths with a high risk will be counted, and the collective impact of those deaths with a small risk of being preventable will be missed.

The second issue with the plan to rank hospitals on the basis of case review is one of small numbers. While challenges in quality measurement associated with small-number phenomena are not new,^6^ ^7^ it is worth considering how small numbers pose challenges in this specific proposed initiative: the emerging policy is to review 2000 hospital deaths every year. Given that previous work with this method found that around 5% of deaths were preventable,^4^ we might expect that about 100 deaths will be identified as preventable every year. One would hope that an equal number of cases were reviewed per hospital to make comparisons fair, and because there are around 160 acute hospitals in England, this equates to less than one death per hospital on average. Statistical theory is not required to realise that this sample will be grossly inadequate in terms of precision and reliability.

An alternative approach might be to use an algorithm designed to identify a set of deaths with a much higher chance of being avoidable, based on routine data (eg, information included in patients’ electronic health records). Even if a highly efficient algorithm could be developed, which selected deaths of which 50% were found to be preventable, assuming 2000 hospital deaths sampled from 160 hospitals as proposed, such an algorithm would still result in hospital ranks based on approximately six potentially preventable deaths per hospital on average. While selecting deaths with a 50% chance of being preventable based on routine data alone is highly unrealistic, even in this scenario, it is very unlikely that the number of observations will be adequate. To better understand the implications, we can consider these data in the context provided by the binomial distribution. If there were 13 cases reviewed per hospital (2080 in total), and on average, 50% of those deaths were flagged as preventable, we would expect a range of observed numbers of preventable deaths across all hospitals just by chance alone. A useful analogy here is one of flipping a coin. One person flipping a coin 13 times may get 10 heads and three tails whereas another person may get only six heads. If each person continued flipping the coin many times, we would expect both to get a head close to half of the time, but when considering only a small number of flips, large variation can be seen.

**Figure 1:**
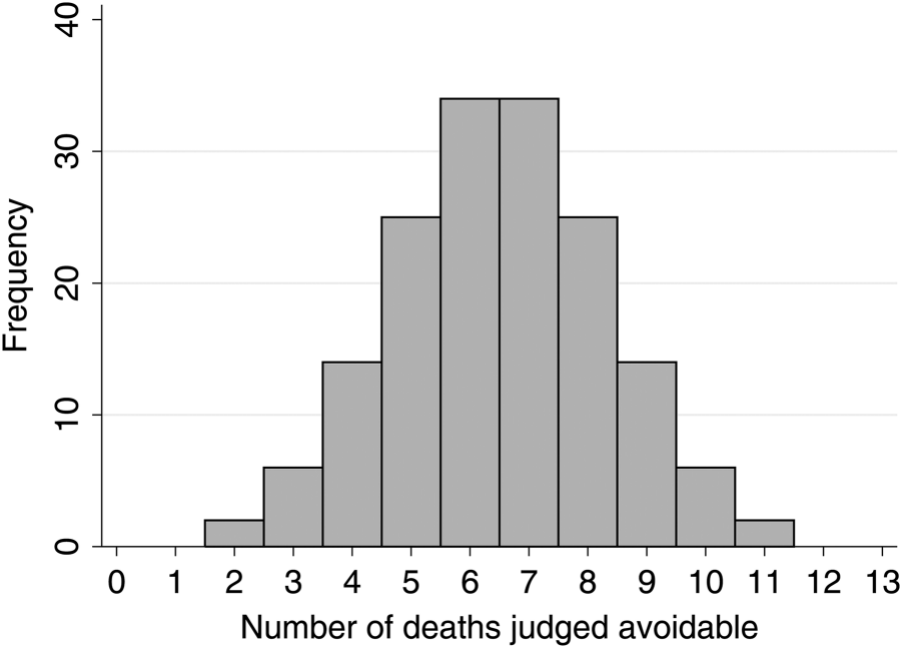
Distribution of the expected number of avoidable deaths for 160 hospitals, each having 13 deaths reviewed, where deaths selected for review have been chosen such that 50% are expected to be avoidable. Even with this unrealistically high probability of preventable deaths, chance variation alone produces substantial variation, with some hospitals apparently having five times the number of preventable deaths as others.

Figure 1 shows the expected distribution for any 1 year in the scenario described above (160 hospitals with 13 deaths reviewed, of which 50% are deemed preventable). This distribution has very large variability. The, apparently, worst hospitals have 5.5 times as many preventable deaths than the, apparently, best hospitals. This, however, is due to chance alone. Additionally, we expect eight hospitals on average to have 10 or more of these 13 cases flagged as preventable even if there is no true variation between hospitals. This illustration is for a typical year, and once every 4 years, we would expect one hospital to have all but one of the cases reviewed flagged as preventable, purely due to chance variability. It should be remembered that this is almost certainly an unrealistic scenario, and performance of any algorithm would almost always be worse than that outlined here. Furthermore, the chance variation illustrated will be further exaggerated when extrapolating back to the entire hospital cohort using an appropriate weighting scheme. Previous simulation studies have also provided concordant evidence.^8^

The two issues outlined here are not without potential cure. The use of a dichotomising cut-off when defining preventable deaths can be overcome by recognising the full range of probabilities and factoring them into calculations. For example, this can be done by preserving the case reviewers’ estimate of preventability. By doing so, the number of deaths that could be avoided can be estimated by multiplying the mean chance of a death being preventable by the total number of hospital deaths. In turn, the issue of small numbers may be addressed simply by reviewing more cases. In order to determine how many cases is enough, one should consider the statistical reliability (also known as rankability) of the resulting metrics.^9–12^ Reliability, in this context, is defined as the proportion of observed variance in hospital scores/metrics that is explained by the true (underlying) variance in hospital performance or, put another way, the proportion of the overall observed variation *not* due to noise. When sample sizes for individual hospitals are small, the uncertainty on those individual hospital scores will artificially inflate the apparent variance between hospitals. When the difference between true and observed variance is small, metrics may be considered reliable; however, there are times when the true variability accounts for only a minority of the observed variability,^12^ and metrics are highly unreliable. It should be noted that reliability depends on sample size and the degree of true variability between hospitals as it is easier to distinguish hospitals when there is larger variation between them. Of course, it may not be feasible or practical to review enough cases to produce reliable ranks.

A related concept worth consideration is the identification of outliers. There are many ways in which this is done, but often hospitals for which there is statistical evidence that their performance differs from the national average are flagged as outliers. Some hospitals are likely to be flagged as outliers even when reliability is low. In such situations (and to a lesser degree when reliability is high), we can observe the apparently paradoxical situation when hospitals flagged as better or as worse than average are not statistically significantly different from some hospitals flagged as average.^13^ This apparent paradox is easily explained when one considers that the hypothesis considered when testing if a hospital is different from the national average is not the same as when testing if a hospital is different to another individual hospital.^14^ It is easier to distinguish a hospital from a national average than it is to distinguish from another hospital. When ranking hospitals, implicit comparisons between individual hospitals are being made, and thus, it is the latter distinction that matters rather than the former. It should also be borne in mind that when reliability is very high, nearly all hospitals will be statistically significantly different from the national average. For such reasons, it is often preferable to account for the real variation between hospitals, and the resulting overdispersion, when identifying outliers.^15^

In conclusion, classifying the performance of English hospitals for avoidable hospital mortality based on a review of 2000 hospital deaths per year will result in both invalid and unreliable rankings. In spite of the limitations of HSMR and similar metrics, they do not suffer from the issues described here. For example, when estimating the HSMR value for a given hospital, no attempt is made to classify individual deaths as preventable or not; rather, the total number of deaths is simply compared with the expected number given the case-mix of patients. Further, given all deaths are counted, small number issues are minimised. Given known methodological limitations of HSMR use, this leaves the improvement community and policy-makers in an uncertain, in fact uncomfortable, place. It should be acknowledged that the very notion of case reviews may be beneficial in itself. For example, it can help to engage hospital leaders on reflecting about their own performance, and incentivise and motivate local improvements. It may also be useful for determining a useful national benchmark.^4^ Therefore, this new initiative may result in quality improvements; it will, however, remain a grossly inadequate measure for judging comparative hospital performance in respect of avoidable mortality.
